# Supplementary material for: Qualitative study exploring barriers and facilitators to progression for female medical clinical academics: interviews with female associate professors and professors
Source: BMJ Open. 2022 Mar 14;12(3):e056364. doi: 10.1136/bmjopen-2021-056364 (PMC8921847; doi:10.1136/bmjopen-2021-056364)
Supplement: Supplementary data [file bmjopen-2021-056364supp001.pdf]

COREQ checklist (from Consolidated criteria for reporting qualitative research (COREQ): a 32-item checklist for interviews and focus groups

No Item Guide questions/description.	Page(s)
Domain 1: Research team and reflexivity	
Personal Characteristics<br>1. Interviewer/facilitator Which author/s conducted the interview or focus group?<br>2. Credentials What were the researcher's credentials? E.g. PhD, MD<br>3. Occupation What was their occupation at the time of the study?<br>4. Gender Was the researcher male or female?<br>5. Experience and training What experience or training did the researcher have?<br>Relationship with participants<br>6. Relationship established Was a relationship established prior to study commencement?<br>7. Participant knowledge of the interviewer. What did the participants know about the researcher? e.g. personal goals, reasons for doing the research<br>8. Interviewer characteristics What characteristics were reported about the interviewer/facilitator? e.g. Bias, assumptions, reasons and interests in the research topic	6-7
Domain 2: study design<br>Theoretical framework<br>9. Methodological orientation and Theory<br>What methodological orientation was stated to underpin the study? e.g. grounded theory,discourse analysis, ethnography, phenomenology, content analysis<br>Participant selection<br>10. Sampling How were participants selected? e.g. purposive, convenience, consecutive, snowball<br>11. Method of approach How were participants approached? e.g. face-to-face, telephone, mail, email<br>12. Sample size How many participants were in the study?<br>13. Non-participation How many people refused to participate or dropped out? Reasons?<br>Setting<br>14. Setting of data collection Where was the data collected? e.g. home, clinic, workplace<br>15. Presence of non-participants Was anyone else present besides the participants and researchers?<br>16. Description of sample What are the important characteristics of the sample? e.g. demographic data, date<br>Data collection<br>17. Interview guide Were questions, prompts, guides provided by the authors? Was it pilot tested?<br>18. Repeat interviews Were repeat interviews carried out? If yes, how many?<br>19. Audio/visual recording Did the research use audio or visual recording to collect the data?<br>20. Field notes Were field notes made during and/or after the interview or focus group?<br>21. Duration What was the duration of the interviews or focus group?<br>22. Data saturation Was data saturation discussed?	5-6

23. Transcripts returned Were transcripts returned to participants for comment and/or correction?	
Domain 3: analysis and findings<br>Data analysis<br>24. Number of data coders How many data coders coded the data?<br>25. Description of the coding tree Did authors provide a description of the coding tree?<br>26. Derivation of themes Were themes identified in advance or derived from the data?<br>27. Software What software, if applicable, was used to manage the data?<br>28. Participant checking Did participants provide feedback on the findings?	7
Reporting<br>29. Quotations presented Were participant quotations presented to illustrate the themes / findings? Was eachquotation identified? e.g. participant number<br>30. Data and findings consistent Was there consistency between the data presented and the findings?<br>31. Clarity of major themes Were major themes clearly presented in the findings?<br>32. Clarity of minor themes Is there a description of diverse cases or discussion of minor themes?	8-19
