# Supplementary material for: Qualitative study exploring barriers and facilitators to progression for female medical clinical academics: interviews with female associate professors and professors
Source: BMJ Open. 2022 Mar 14;12(3):e056364. doi: 10.1136/bmjopen-2021-056364 (PMC8921847; doi:10.1136/bmjopen-2021-056364)
Supplement: Supplementary data [file bmjopen-2021-056364supp002.pdf]

Interview guide

1. Can you tell me about your decision to apply for Clinical Associate Professor?
2. Have you had to overcome any barriers to get to your current position? If so, how did you do that?
3. What about facilitators: are there any examples of positive experiences that you have encountered that helped with career progression? Do you have any ideas or thoughts on further support?
4. Do you have any caring responsibilities? If so, who do you share those caring responsibilities with, and how?
5. Do you currently, or would you like to, work less than full time for any reason? Do you experience or anticipate any problems with working less than full time?
6. If you have caring responsibilities, what types of support have you found most helpful in helping you to combine family life and a clinical academic career?
7. What support would you like to see more of?
8. Have discussions as part of your joint clinical academic appraisal helped in addressing barriers to progression? If so, how?
9. What do you think the university could do to improve the numbers of female Clinical Associate Professors, Clinical Senior Lecturers and Clinical Professors?
10. What advice would you give to women who are thinking of applying for a Clinical Associate Professor post?
11. Do you have any specific advice for women with caring responsibilities who are thinking of applying for a Clinical Associate Professor post?
12. How do current practices and procedures help or hinder women along the clinical academic pathway?
